# Mitochondrial Dysfunction and Alzheimer’s Disease: Role of Microglia

**DOI:** 10.3389/fnagi.2020.00252

**Published:** 2020-08-20

**Authors:** Ishan Agrawal, Sushmita Jha

**Affiliations:** Inflammation, Immunity and Tumour Biology Laboratory, Department of Bioscience and Bioengineering, Indian Institute of Technology (IIT) Jodhpur, Jodhpur, India

**Keywords:** microglia, mitochondria, ROS, amyloid-β, neurodegeneration

## Abstract

In 1907, Alois Alzheimer observed, as he quoted, development of “numerous fibers” and “adipose saccules” in the brain of his diseased patient Auguste Deter. The neurodegenerative disease became known as Alzheimer’s disease (AD) and is the most common cause of dementia worldwide. AD normally develops with aging and is mostly initiated because of the imbalance between the formation and clearance of amyloid-β (Aβ). Formation of neurofibrillary tangles (NFTs) of hyperphosphorylated tau is another hallmark of AD. Neuroinflammation plays a significant role in the development and pathology of AD. This chapter explores the role of mitochondrial dysfunction in microglia in case of AD. Mitochondrial oxidative stress in microglia has been linked to the development of AD. Elevated generation of reactive oxygen species (ROS) and loss of mitochondrial membrane potential through various mechanisms have been observed in AD. Aβ interacts with microglial receptors, such as triggering receptor expressed in myeloid cells 2 (TREM2), activating downstream pathways causing mitochondrial damage and aggravating inflammation and cytotoxicity. Fibrillar Aβ activates nicotinamide adenine dinucleotide phosphate (NADPH) oxidase in microglia leading to elevated induction of mitochondrial ROS which further causes neurotoxicity. Elevated ROS in microglia causes activation of inflammatory and cell death pathways. Production of ATP, regulation of mitochondrial health, autophagy, and mitophagy in microglia play significant roles in the AD pathology. Understanding microglial physiology and mitochondrial dysfunction will enable better therapeutic interventions.

## Introduction

In 1907 at the state asylum in Frankfurt, Germany, Aloysius “Alöis” Alzheimer meticulously described the symptoms of his 51-year-old patient Auguste Deter which included memory loss and other cognitive impairment. After the death of Auguste Deter, Alzheimer histologically analyzed her brain with silver staining and observed the development of neurofibrillary tangles (NFTs), neuritic plaques, and amyloid angiopathy. These observations became the hallmark of the disease, which came to be known as Alzheimer’s disease (AD; Bondi et al., 2017). AD is the most common cause of dementia worldwide. It accounts for about 80% of all diagnosed cases (Weller and Budson, 2018). Every 3 s, an individual is diagnosed with dementia. About 50 million people around the world were living with dementia (in 2018), and two-thirds of them had AD. This population is estimated to become 152 million by 2050 (Patterson, 2018). AD largely affects the aging population. The effect of AD on the global economy is undeniable, as dementia costs approximately US$ 1 trillion annually (in 2018), which is expected to double by 2030 (Patterson, 2018; Weller and Budson, 2018). In the United States, AD claims more lives than prostate and breast cancer combined and is the number one killer in Wales and England (Patterson, 2018). Rising cases and high mortality of AD highlight the need for a more comprehensive study to improve patient care and future therapeutics. While the genetic, environmental, and immune aspects of AD are being studied extensively, this chapter will focus on the role of mitochondrial dysfunction in microglia in AD pathology.

## Alzheimer’s Disease

### Genetic Factors

AD is categorized into two forms: sporadic and familial. Almost 99% cases of AD are sporadic, i.e., the exact cause of onset in unknown (Wang et al., 2017). The most definitive cause remains aging. Symptoms generally manifest around the age of 60–65 years. Sporadic AD (SAD) or late-onset AD (LOAD) is most likely driven by genetic as well as environmental factors (Bondi et al., 2017; Lane et al., 2018). The major genetic factor for SAD is the apolipoprotein E (*APOE*) gene which has three variants: *e2*, *e3*, and *e4*. APOE protein is responsible for packaging and carrying cholesterol and other fats through the bloodstream. Every individual inherits one of the three forms of APOE gene from each parent, most commonly the *e3* form. Inheriting *e4* significantly increases the risk of developing AD with respect to *e3*, whereas inheriting *e2* might reduce the risk (Alzheimer’s Association, 2018). People inheriting one copy of *e4* are three times more likely to develop AD compared with people inheriting two *e3* forms, whereas the ones having two copies of *e4* are 8–12 times more at risk. However, having *e4* form of the APOE gene does not guarantee the development of AD. In the last decade, genome-wide association studies and whole genome sequencing studies have identified several novel genetic factors like triggering receptor expressed in myeloid cells 2 (TREM2), complement C3b/C4b receptor 1 (CR1), CR1 (complement C3b/C4b receptor 1), CD33, and ABCA7 that are associated with a high risk of LOAD (Zheng et al., 2018). Many of these are specifically or preferentially expressed in microglia, TREM2 being the most studied (Hansen et al., 2018). TREM2 is a microglial cell surface receptor central to phagocytosis, chemotaxis, survival, and proliferation of microglia (Carmona et al., 2018). The TREM2 loss-of-function mutation R47H results in a two- to four-fold increase in the risk of AD similar to the risk associated with inheriting one copy of the *e4* variant of APOE (Gratuze et al., 2018). TREM2 binds to all three isoforms of APOE and other lipoprotein particles (Gratuze et al., 2018; Zheng et al., 2018). The TREM2–APOE binding is reduced in the case of disease-associated mutations of TREM2 which is believed to affect the pathology of AD (Yeh et al., 2016; Jay et al., 2017; Zheng et al., 2018). APOE–TREM2 crosstalk results in the transcriptional shift in microglia in AD which induces the loss of homeostatic capability of microglia and consequent neurodegeneration (Krasemann et al., 2017; Ennerfelt and Lukens, 2020).

Familial AD (FAD) is characterized by the development of symptoms before the age of 60 or sometimes even 55. If one or more than one member in the family is diagnosed with AD, then the next generation or even siblings are more likely to develop the disease compared with individuals with no family history of AD (Alzheimer’s Association, 2018). FAD is associated with the mutation in three proteins: amyloid precursor protein (APP), presenilin 1 (PSEN1), and presenilin 2 (PSEN2). All of these genetic factors associated with SAD and FAD are involved in the imbalance between generation and clearance of amyloid-β (Aβ) plaques (Baranello et al., 2015).

### Pathology

Research in the last two decades has provided compelling, though not definitive, evidence that the major cause of AD is the development and accumulation of Aβ plaques and NFTs. This hypothesis is referred to as the *amyloid hypothesis* or *amyloid cascade hypothesis* (Querfurth and LaFerla, 2010; De Strooper and Karran, 2016; Lane et al., 2018). Aβ plaques accumulate outside cells whereas NFTs are formed inside neurons. The accumulation of Aβ plaques and NFTs leads to neurodegeneration causing loss of neurons and synapses. Aβ is produced by the sequential processing of APP by a β-secretase called beta-site amyloid precursor protein–cleaving enzyme 1 (BACE1), and γ-secretase a protein complex which has PSEN1 and PSEN2 as its catalytic subunits (Querfurth and LaFerla, 2010; Scheltens et al., 2016; Sanabria-Castro et al., 2017). Processing of APP leads to the production of 37–43 amino acid Aβ peptides among which Aβ_42_ is the most toxic. Aβ peptides are resistant to proteolytic degradation and an imbalance between generation and clearance of Aβ leads to its accumulation and development of Aβ plaques. Aβ can spontaneously self-aggregate and can coexist in multiple physical forms. One of the forms contains oligomers of 2–6 peptides that further coalesce to form intermediate assemblies (Querfurth and LaFerla, 2010). Aβ can also form fibrils that assemble into β-pleated sheets resulting in insoluble fibers that make advance amyloid plaques (Querfurth and LaFerla, 2010; Sanabria-Castro et al., 2017). Intermediate amyloids and soluble oligomers are considered as the most neurotoxic forms of Aβ. Aβ peptides are degraded by insulin-degrading enzyme (IDE) and neprilysin in the brain (Kanemitsu et al., 2003; Querfurth and LaFerla, 2010). In case of both aging and AD, the expression of IDE and neprilysin decreases, causing an imbalance in the homeostasis of Aβ (Querfurth and LaFerla, 2010; Sanabria-Castro et al., 2017).

Another hallmark of AD is the production and accumulation of NFTs that are primarily composed of hyperphosphorylated tau protein. Tau is a soluble protein present in neurons that plays an important role in maintaining the stability of microtubules (Iqbal et al., 2005). Microtubules provide structural support to neurons and are responsible for healthy axonal transport and neuronal growth (Querfurth and LaFerla, 2010; Sanabria-Castro et al., 2017). Hyperphosphorylation of tau makes it insoluble, reduces its affinity to microtubules and makes it self-associate into paired helical filamentous structures. Hyperphosphorylation also makes tau resistant to degradation by calcium activated neutral proteases, further reducing its turnover with respect to normal tau (Wang et al., 1995; Iqbal et al., 2010). Similar to Aβ plaques, intermediate aggregation of the hyperphosphorylated tau is toxic and leads to impaired cognition (Querfurth and LaFerla, 2010; Sanabria-Castro et al., 2017). In the pathology of AD, alterations in tau protein are generally considered to be downstream of Aβ, but in some cases, mutation in tau gene leads to dementia without amyloid plaques raising the possibility that Aβ and tau plaques act independently or even in parallel to enhance each other’s toxicity (Scheltens et al., 2016).

Owing to the direct involvement of Aβ and tau plaques in AD pathology, recent studies have elucidated a central role of inflammation in the pathophysiology of AD (Kinney et al., 2018). Microglia are the brain’s resident immune cells and play a central role in AD-related inflammation. Microglia aggregate around plaques and clear them by phagocytosis. The process is more efficient if the plaques are complexed with low-density lipoproteins, apoE and clusterin. Aβ interacts with microglia *via* CD 36 and heterodimer of toll-like receptor 4 and 6. This interaction seems to activate the NLRP3 inflammasome which results in the secretion of IL-1β (Stewart et al., 2010; Gold and El Khoury, 2015). Prolonged expression of these cytokines is cytotoxic and contributes to disease pathology (Hansen et al., 2018).

## Mitochondria in Neurodegeneration and Alzheimer’s Disease

Mitochondria are defined as the powerhouse of the cell. They are responsible for ATP generation, reactive oxygen species (ROS) formation, intracellular calcium homeostasis, and apoptosis (Flannery and Trushina, 2019). Mitochondrial dynamics of fusion, fission, turnover, and transport is governed by energy demand as well as environmental stimuli (Chen and Chan, 2009; Flannery and Trushina, 2019). Mitochondria can multiply through fission and the number can reduce by the process of mitochondrial fusion. Defective mitochondria are removed by a well-organized process called mitophagy (Cenini and Voos, 2019; Flannery and Trushina, 2019). The brain demands high metabolic energy to function; therefore, healthy mitochondria are a prerequisite for a healthy brain. In neurons, mitochondria are needed for energy metabolism, buffering of calcium concentration for fundamental cellular processes, neurotransmission, and for membrane potential generation (Cenini and Voos, 2019). It is for these reasons that the demand and turnover of mitochondria in neurons is high. Mitochondrial dysfunction in neurons has been considered an important factor in all neurodegenerative diseases including AD. Aβ has been reported to damage mitochondria and interfere with its functioning (Lezi and Swerdlow, 2012). “Mitochondrial cascade hypothesis” for AD was introduced in 2004 which considers mitochondrial dysfunction as the prerequisite that further triggers the events leading to LOAD (Cenini and Voos, 2019; Flannery and Trushina, 2019). Impaired oxidative phosphorylation (OXPHOS), decreased production of ATP, and increase in oxidative stress are well documented in AD (Wang et al., 2014; Flannery and Trushina, 2019). As oxidative stress increases with the defect in OXPHOS, an increase in Aβ and phosphorylated tau has been reported (Flannery and Trushina, 2019). Imbalance in mitochondrial dynamics of fission and fusion also occur in AD leading to irregular mitochondrial distribution in neurons. Mitochondrial biogenesis is also impaired in AD (Cenini and Voos, 2019). Many neurological diseases are related to hereditary or non-hereditary changes in mitochondria that may lead to inflammation. Damage to mitochondria leads to deficiency in energy production, oxidative stress, and generation of mitochondria-derived damage-associated molecular patterns, causing inflammation and neuronal damage (Mathew et al., 2012; Wilkins et al., 2017). Recently, mitochondrial dysfunction in microglia has also been reported to play a significant role in the pathogenesis of AD and other neurological disorders (Picone et al., 2014; Cenini and Voos, 2019; Flannery and Trushina, 2019). Studying mitochondrial dysfunction in microglia is an emerging and promising field of study for understanding the energy dynamics and pathology of AD. This chapter will elucidate the effect of mitochondrial dysfunction in AD with a focus on microglia.

### Microglia and Mitochondrial Dysfunction in AD

Microglia are the resident immune cells of the brain. They are critical to immunity and homeostasis in the central nervous system. Dysregulated microglial function has been related to inflammatory, autoimmune, and infectious diseases as well as Alzheimer’s and Parkinson’s diseases. Aβ activates microglia leading to secretion of proinflammatory cytokines such as IL-1β, IL-6, and TNF-α (Mosher and Wyss-Coray, 2014). Aβ is related to increased expression of nicotinamide adenine dinucleotide phosphate (NADPH), inducible nitric oxide synthase (iNOS), and ROS that along with proinflammatory cytokine release leads to sustained inflammation and neuronal damage (Mosher and Wyss-Coray, 2014; von Bernhardi et al., 2015).

#### TREM2 and Mitochondrial Dysfunction

Microglia respond to Aβ through phagocytosis and through receptors present on them. Complement receptors, Fc receptors, toll-like receptors (TLRs), receptor for advanced glycosylation end products (RAGE), and triggering receptor expressed by myeloid cells 2 (TREM2) are some of the receptors involved (Doens and Fernández, 2014; Fu et al., 2014). Recently, Ulland et al. (2017) reported that TREM2 plays a role in maintaining metabolic fitness of microglia in 5×FAD mouse model of AD. The authors measured the ratio of lipidated LC3II (microtubule-associated protein 1 light chain 3) to non-lipidated LC3I in microglia of 5×FAD mice. Lipidated LC3 is called LC3II and is used as a marker for detecting autophagic vesicles (Tanida et al., 2008). The authors found that microglia from 5×FAD mice that lack TREM2 had high lipidated LC3II to non-lipidated LC3I ratio and increased number of autophagic vesicles than microglia from the 5×FAD wild-type mice (Ulland et al., 2017). Increased number of autophagic vesicles were also observed in AD patients having TREM2 risk variants R47H and R62H (Jin et al., 2014; Ulland et al., 2017). The number of mitochondria and ATP level were less in *TREM2*^−/−^ microglia from 5×FAD mice. The authors reported that TREM2 deficiency leads to impaired mammalian target of rapamycin (mTOR) signaling pathway, which plays a major role in regulation of autophagy and increases autophagy. Decreased phosphorylation of mTOR effector molecules 4EBP1 (eukaryotic translation initiation factor 4E binding protein 1, a translational repressor), Akt (protein kinase B, a serine/threonine kinase), and NDRG1 (N-myc downstream regulated gene 1, regulates cell growth, differentiation, and hormone responses) was observed in *TREM2*^−/−^ microglia from 5×FAD mice. Increasing the supply of ATP by treating *TREM2*^−/−^ mice with cyclocreatine, an analog of creatine that can supply ATP, improved microglial metabolism, microglial response to Aβ, and regulated autophagy. The relation between cellular pathways affected by TREM2 and mitochondrial health in microglia needs further investigation. However, it was evident that TREM2 is needed by microglia to respond to Aβ in an energetically sustainable way (Ulland et al., 2017). Pan et al. saw that sodium rutin (NaR), a sodium salt of natural flavonoid rutin (quercetin-3-rutinoside), can also reduce Aβ pathology in TREM2-deficient mice by significantly enhancing the ATP production. NaR treatment increased the microglial oxygen consumption rate which indicated an enhanced mitochondrial OXPHOS (Pan et al., 2019). The authors observed that biotin–rutin is getting localized to mitochondria, suggesting NaR may directly affect mitochondrial metabolism. Treatment with rotenone/antimycin A, which reduces OXPHOS, reduced the phagocytosis of Aβ by microglia. On the other hand, galactose treatment, which enhances OXPHOS, increased Aβ phagocytosis. TREM2 and ATP both are very important factors for the phagocytosis of Aβ by microglia. Treatment of microglia with NaR enhanced TREM2 expression and rescued TREM2-deficient microglia from energy deficits through OXPHOS. It may be possible that prolonged treatment of *TREM2*^−/−^ mice with NaR may lead to increased phagocytosis of Aβ and hence reduction in AD pathology (Pan et al., 2019). TREM also affects tau pathology in AD. TREM2 deficiency and disease-associated TREM2 have been linked to increased tauopathy (Bemiller et al., 2017). Leyns et al. (2019) investigated TREM2 in correlation with tau and Aβ and found that TREM2-R47H and TREM2 deficiency increased the chances of tau seeding and spread in neurons in the vicinity of Aβ plaques. However, in an independent study, Leyns et al. (2017) reported that impaired TREM2 signaling in microglia reduces neuroinflammation and neurodegeneration. Both studies used different mouse models which may explain the varying findings (Leyns et al., 2017, 2019). The level of soluble TREM2, TREM2 secreted from the microglia, has also been reported to correlate with total and phosphorylated tau in AD patients’ cerebrospinal fluid (Piccio et al., 2016). Links between TREM2 and tau pathology have been investigated in few studies but not in association with mitochondrial dysfunction in microglia (Bemiller et al., 2017; Gratuze et al., 2018). Association between TREM2, tau, and mitochondrial dysfunction needs further investigation and may reveal a better understanding of energy dynamics of AD.

#### Mitophagy in Microglia

Microglial mitophagy plays a significant role in controlling neuroinflammation in AD. Phagocytosis and clearance of Aβ depends on mitochondrial health. It has been observed that the efficiency of microglia to clear Aβ plaques reduces chronic inflammation and mitochondrial dysfunction. Restoring mitochondrial function and mitophagy in microglia helps reduce neuroinflammation and is therefore neuroprotective (Lautrup et al., 2019). Fang et al. (2019) observed that there was an increase in damaged mitochondria in APP/PS1 mice, mouse models for AD, and a 60% reduction in mitophagy in the hippocampus of these mice with respect to WT mice. The authors treated APP/PS1 mice with mitophagy inducers urolithin A (UA) or actinonin (AC) and found that this increased mitophagy and decreased the number of damaged mitochondria. There was also an increase in phagocytosis of Aβ. Microglia reduced the number and lengths of process indicating their shift toward phagocytic morphology. In addition, the expression of CD68, an engulfment-associated protein, increased in the microglia of treated mice. Increase in phagocytosis might have been caused by an increase in healthy mitochondrial population as phagocytosis is an energy-intensive process. Interestingly, the levels of proinflammatory cytokines IL-6 and TNF-α also reduced in UA- and AC-treated mice. There was a fourfold increase in IL-10 levels in the hippocampal tissue of treated mice (Fang et al., 2019). Previously, it has been reported that IL-10 increases mitophagy in macrophages (Ip et al., 2017; Lautrup et al., 2019). It might be possible that in the case of UA and AC treatment, increased IL-10 further promotes mitophagy in microglia, though it needs further exploration. PTEN-induced kinase 1 (PINK1) is one of the major proteins involved in mitophagy. Knocking down PINK1 in isolated microglia from APP/PS1 and WT mice increased the expression of TNF-α and eliminated the effect of UA treatment. APP/PS1 mice also had an increased expression of NLRP3, IL-1β, and cleaved caspase 1 which was significantly reduced by UA treatment (Fang et al., 2019). Suppressing proinflammatory cytokines has been reported previously to improve microglial phagocytosis in APP/PS1 model. Microglia isolated from NLRP3 and caspase 1 knockout mice show an increase in phagocytosis (Heneka et al., 2013). Lei et al. saw that treatment of BV-2 microglia with mitochonic acid 5 (MA-5), a mitophagy inducer, increases mitochondrial quality, reduces neuroinflammation and mitochondrial apoptosis, and neutralizes ROS overproduction (Lei et al., 2018).

#### Apoptosis and Mitochondria

Aβ may cause apoptosis in microglia. Pifithrin-α treatment along with Aβ significantly reduces the number of apoptotic rat primary microglia. Pifithrin-α is a p53-dependent transcriptional activation inhibitor. Aβ treatment increases cytoplasmic p53 in microglia (Davenport et al., 2010). p53 and Aβ have been reported to localize to mitochondria and induce apoptosis in neuronal cells (Cha et al., 2012; Dai et al., 2016; Maj et al., 2017). However, whether the same phenomena occur in microglia also needs further investigation. Xie et al. (2014) saw that inhibition of mitochondrial fission reduces Aβ-induced microglial apoptosis. Mitochondrial division inhibitor 1 (mdivi-1) inhibits mitophagy by selectively inhibiting a mitochondrial fission protein called dynamin-related protein 1 (Drp1). Treatment of BV-2 mouse microglia cell line with mdivi-1 reduced Aβ-induced apoptosis. Pretreatment with mdivi-1 also restored the mitochondrial membrane potential in Aβ-treated BV-2 cells and suppressed cytochrome *c* (cytc) release and activation of caspase 3 (Xie et al., 2014). Mitochondrial calcium concentration is also important for healthy mitochondria and microglia. In an independent study, Xie et al. (2017) reported that Aβ causes an increase in mitochondrial calcium concentration in microglia through mitochondrial calcium uniporter (MCU). Inhibition of MCU by Ru360 inhibitor reduced the excessive calcium uptake and inhibited apoptosis in Aβ-treated BV-2 and mouse primary microglia. The authors further analyzed that the level of C/-EBP homologous protein (CHOP), an endoplasmic reticulum stress protein related to mitochondrial ROS production, was increasing in Aβ cells which may be leading to cellular cytotoxicity. Ru360 treatment inhibited the production of mitochondrial ROS and reduced the level of CHOP, leading to enhanced cell viability (Xie et al., 2017). These results suggested that maintaining a healthy population of mitochondria is crucial for microglial apoptosis.

#### P2X_7_R and Mitochondrial Dysfunction

Chiozzi et al. (2019) showed that Aβ-induced mitochondrial toxicity in microglia is P2X_7_ receptor (P2X_7_R) dependent (Sanz et al., 2009; Chiozzi et al., 2019). Treatment of microglia with Aβ causes NFκB activation, NLRP3 activation, and mitochondrial toxicity. In primary mouse microglia and N13 microglial cell line treated with Aβ for 24 h, there was a significant increase in mitochondrial potential, whereas the variation in mitochondrial potential was much less, however significant, in microglia isolated from *P2X7R*^−/−^ mice and N13 cells with reduced expression of P2X_7_R (N13R). Aβ also induced an increase in ROS production in N13 cells that was P2X_7_R dependent (Chiozzi et al., 2019). Aβ-induced mitochondrial toxicity is mediated by cytc (Kim et al., 2002). Aβ-treated N13 cells showed a significant increase in cytc release from mitochondria and accumulation in cytosol. Moreover, 30% lower release of cytc was seen in N13R cells. Release of cytc causes apoptosis in microglia which contributes to AD pathology. The authors also observed that nimodipine, a calcium channel blocker, was able to inhibit Aβ-induced microglial inflammatory response and rescue mitochondria from Aβ-induced toxicity (Chiozzi et al., 2019). Along with mitochondrial hyperpolarization in mitochondria in response to Aβ as reported by Chiozzi et al. (2019), mitochondrial depolarization in microglia also needs to be studied for AD. It needs more detailed study to conclude how Aβ is affecting the membrane potential of mitochondria in microglia and its subsequent effects.

#### Copper and Mitochondrial Dysfunction in AD

Copper homeostasis is affected in AD. Elevated concentration of copper [Cu(II)] has been observed in the cerebrospinal fluid of AD patients (Hozumi et al., 2011). Yu et al. (2015) saw that Cu(II)-Aβ1–40 complex activates BV-2 microglial cell lines which produce TNF-α and nitric oxide, leading to neurotoxicity. Conditioned medium from rat primary microglia treated with Cu(II)-Aβ1–40 was also causing neurotoxicity. Interestingly, it was seen that Cu(II)-Aβ1–40 complex caused an increase in mitochondrial ROS production in BV-2 microglia cell lines. Treatment of BV-2 cell line with *N*-acetyl-cysteine (NAC), which is a ROS scavenger, reduces the production of TNF-α and nitric oxide. Conditioned media from NAC-treated primary rat microglia in the presence of Cu(II)-Aβ1–40 complex was also not neurotoxic (Yu et al., 2015). Although this highlights the possibility that copper may play a role in mitochondrial dysfunction in microglia in case of AD, further experimental confirmation is needed. Metal ions play a significant role in homeostatic neurological functions and disorders (Liu et al., 2019). Their role with respect to mitochondrial dysfunction in microglia and neurological disorders needs detailed investigation.

## Conclusion

**Figure 1:**
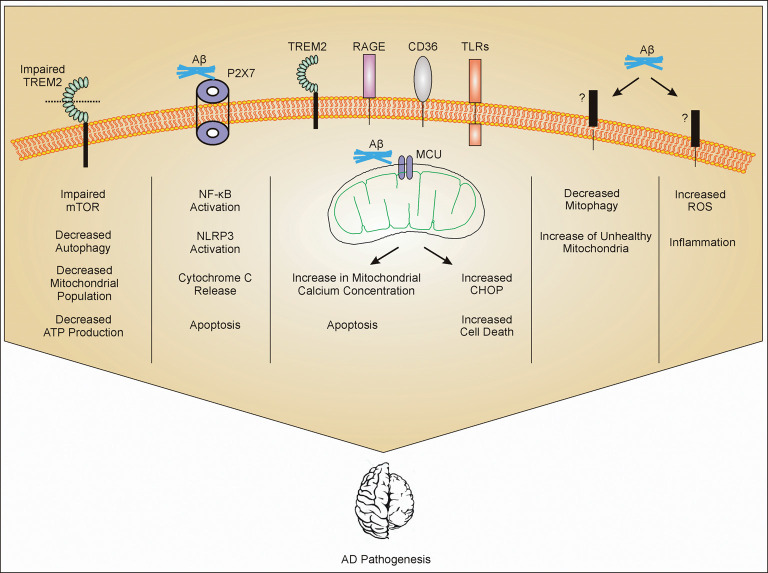
Mitochondrial dysfunction in microglia. Amyloid-β (Aβ) interacts with different receptors present on microglia. Impaired triggering receptor expressed in myeloid cells 2 (TREM2) leads to impaired mammalian target of rapamycin (mTOR) pathway. Impaired mTOR pathway increases autophagy and decreases population of mitochondria which further decrease the production of ATP. Aβ interaction with P2X_7_ activates NF-κB and leads to activation of NLRP3 and release of cytochrome *c* (cytc) from mitochondria (mechanism not clear) and apoptosis. Aβ gets phagocytosed through different receptors such as TREM2, toll-like receptors (TLRs), receptor for advanced glycosylation end products (RAGE), and CD36, and internalized Aβ interacts with mitochondrial calcium uniporter (MCU) on mitochondria leading to cell cytotoxicity. Through unknown receptors (?), Aβ decreases mitophagy and also increases reactive oxygen species (ROS) production. All these mechanisms contribute to Alzheimer’s Disease (AD) pathogenesis through inflammation or other mechanisms which are still under investigation.

**Figure 2:**
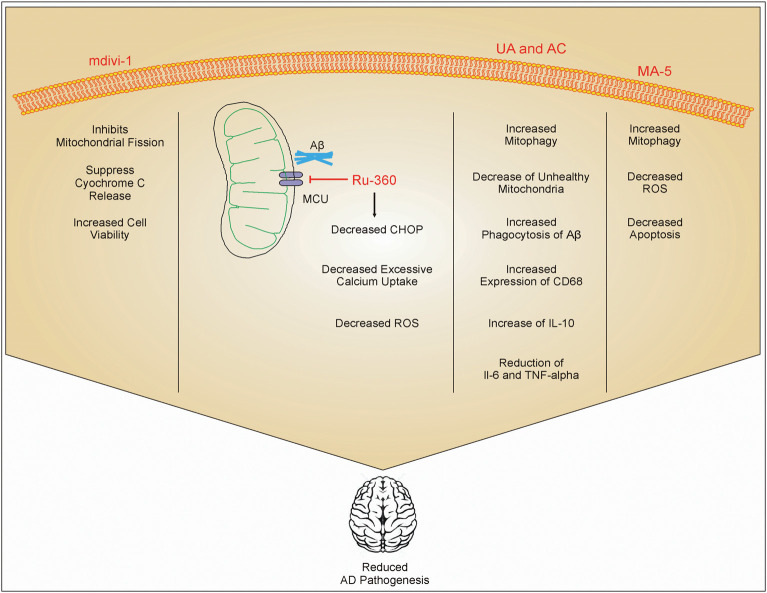
Role of different drugs in improving AD pathology. mdivi-1 inhibits mitochondrial fission and inhibits the release of cytc, which in turn increases cell viability. Ru-360 decreases excessive calcium uptake by mitochondria and reduces the expression of C/-EBP homologous protein (CHOP) on the endoplasmic reticulum. This inhibits ROS production. Urolithin A (UA) and actinonin (AC) increases phagocytosis of Aβ and increases mitophagy which reduces the number of damaged mitochondria inside cells. UA and AC also increase the expression of IL-10 and inhibit IL-6 and TNF-α. Mitochonic acid 5 (MA-5) also increases mitophagy and decreases the production of ROS. These drug effects enhanced cell viability, and reduced inflammation and AD pathology.

Aβ induces inflammatory response in AD through various mechanisms (Figure 1). Microglial receptors like TREM2, Fc receptors, formyl peptide receptors, complement receptors, scavenger receptors, RAGE, toll-like receptors (TLRs), NLRP3 inflammasome, and others play a significant role in Aβ-induced inflammation (Doens and Fernández, 2014). Although TREM2 remains the most studied receptor in AD, other receptors need to be investigated as potential regulators of disease pathophysiology and outcome (Zheng et al., 2018). Although the effects of metal ions, such as zinc and iron, on neurons have been studied in AD, the relationship between mitochondrial dysfunction in microglia and metal ions remains largely underexplored (Scott and Orvig, 2009; Sensi et al., 2011; Liu et al., 2019). It is clear that mitochondrial dynamics in microglia also plays a significant role in the progression of AD (Flannery and Trushina, 2019). However, the effect of various factors related to AD on mitochondrial health in microglia is still underexplored. Most of the drugs regulating microglia-related AD pathology are still in experimental stages (Figure 2). The effect of mitochondrial ROS generation on mitochondrial health and vice versa in AD is an interesting area that needs further exploration. Importantly, the role of hyperphosphorylated tau in mitochondrial health in microglia is still unexplored. Mitochondrial dysfunction in microglia is a promising area of research to better understand energy dynamics of AD. It is evident that mitochondrial dysfunction in neurons play a significant role in AD (Picone et al., 2014). However, along with the increasing number of studies, it is becoming clear that mitochondrial dysfunction in microglia contributes significantly to the pathogenesis and progression of AD. Further studies in the area may lead to therapeutic interventions to delay onset or progression of AD.

## Author Contributions

IA prepared the initial article draft and images. SJ provided content expertise, overall direction, and edited and reviewed the article. All authors contributed to the article and approved the submitted version.

## Conflict of Interest

The authors declare that the research was conducted in the absence of any commercial or financial relationships that could be construed as a potential conflict of interest.
